# Robot-assisted excision of cervical cystic hygroma through a retroauricular hairline approach: a case report

**DOI:** 10.1186/s13256-016-0929-0

**Published:** 2016-06-09

**Authors:** Frank Cheau-Feng Lin, Tsung-Lin Yang, Min-Che Tung, Stella Chin-Shaw Tsai

**Affiliations:** 1 Department of Surgery, Chung Shan Medical University Hospital, Taichung, Taiwan, ROC; 2 Department of Otolaryngology, National Taiwan University Hospital and College of Medicine, National Taiwan University, Taipei, Taiwan, ROC; 3 Department of Surgery, Tungs’ Taichung MetroHarbor Hospital, Taichung, Taiwan, ROC; 4 Department of Otolaryngology, Tungs’ Taichung MetroHarbor Hospital, 699, Sec.8, Taiwan Boulevard, Taichung, 43503 Taiwan, ROC

**Keywords:** Cystic hygroma, Minimally invasive surgery, Robotic-assisted surgery

## Abstract

**Background:**

Cystic hygroma is a rare benign abnormality of the lymphatic system generally occurring in young children less than 2 years old. The standard transcervical surgical treatment of cystic hygroma may often leave a permanent scar in the neck region.

**Case presentation:**

We report a case of cystic hygroma in a 19-month-old Asian baby girl successfully treated with robot-assisted excision through a hairline neck-lift approach. We present the use of the Yang’s retractor as an instrumental advancement to this surgical approach.

**Conclusions:**

Treatment options for cystic hygroma may be surgical or nonsurgical. We report a case of cystic hygroma in a 19-month-old child successfully treated with robot-assisted excision through a small concealed retroauricular hairline approach. This is the first report in the medical literature of treating cystic hygroma with a minimally invasive robot-assisted excision via a small, concealed, hairline incision.

**Electronic supplementary material:**

The online version of this article (doi:10.1186/s13256-016-0929-0) contains supplementary material, which is available to authorized users.

## Background

Cystic hygroma, also known as macrocystic lymphangioma, is a rare congenital malformation of the lymphatic system, the majority of which are diagnosed by the end of the second year of life [1]. Cystic hygroma can affect the child’s appearance, swallowing, and even the airway patency. While surgery is the mainstay treatment modality for cystic hygroma [2], it often leaves a visible scar on the neck. We report a case of cystic hygroma occurring in a child after trauma treated with robot-assisted excision through a retroauricular hairline approach.

## Case presentation

**Fig. 1:**
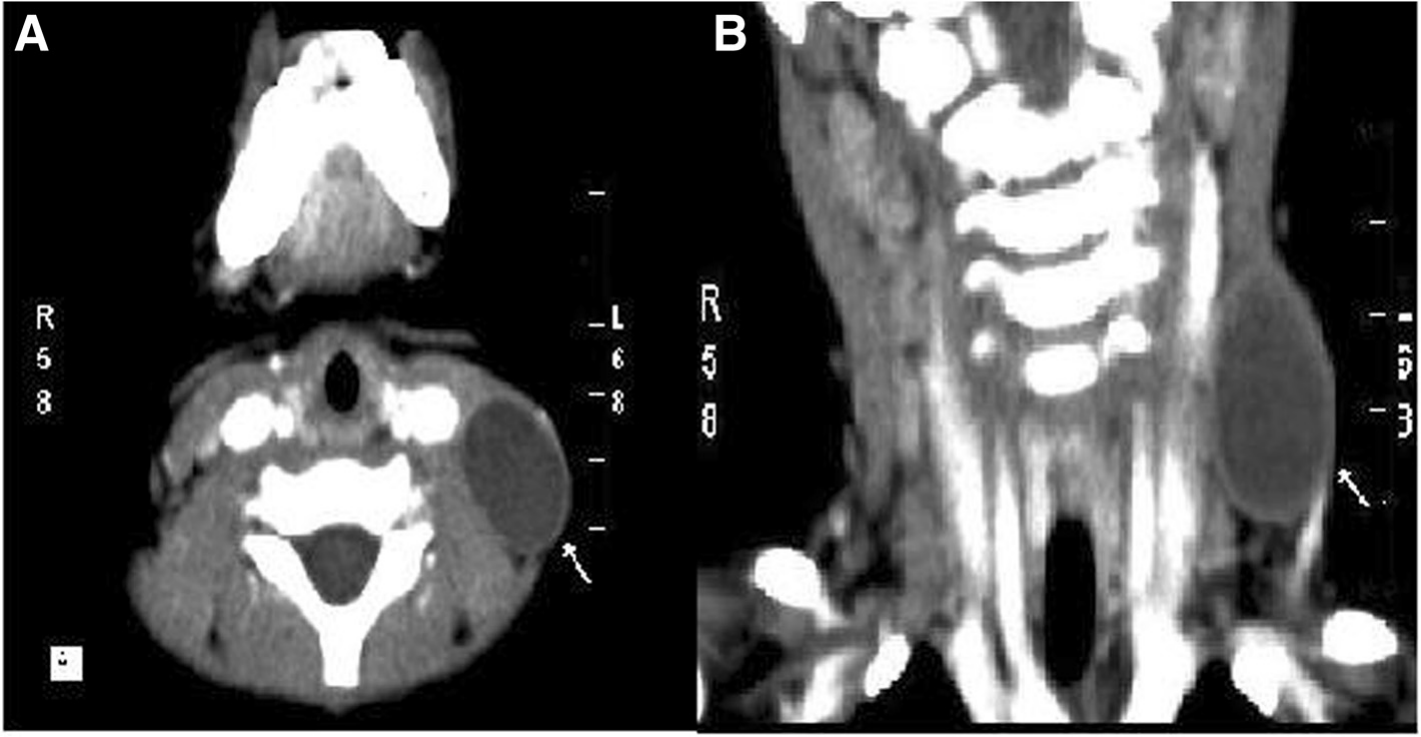
Neck computed tomography scan with contrast enhancement. **a** axial scan and **b** coronal scan; there is a 3 cm × 3 cm sized, well-encapsulated homogenous low-density mass in the left posterior triangle neck area (*arrows*)

**Fig. 2:**
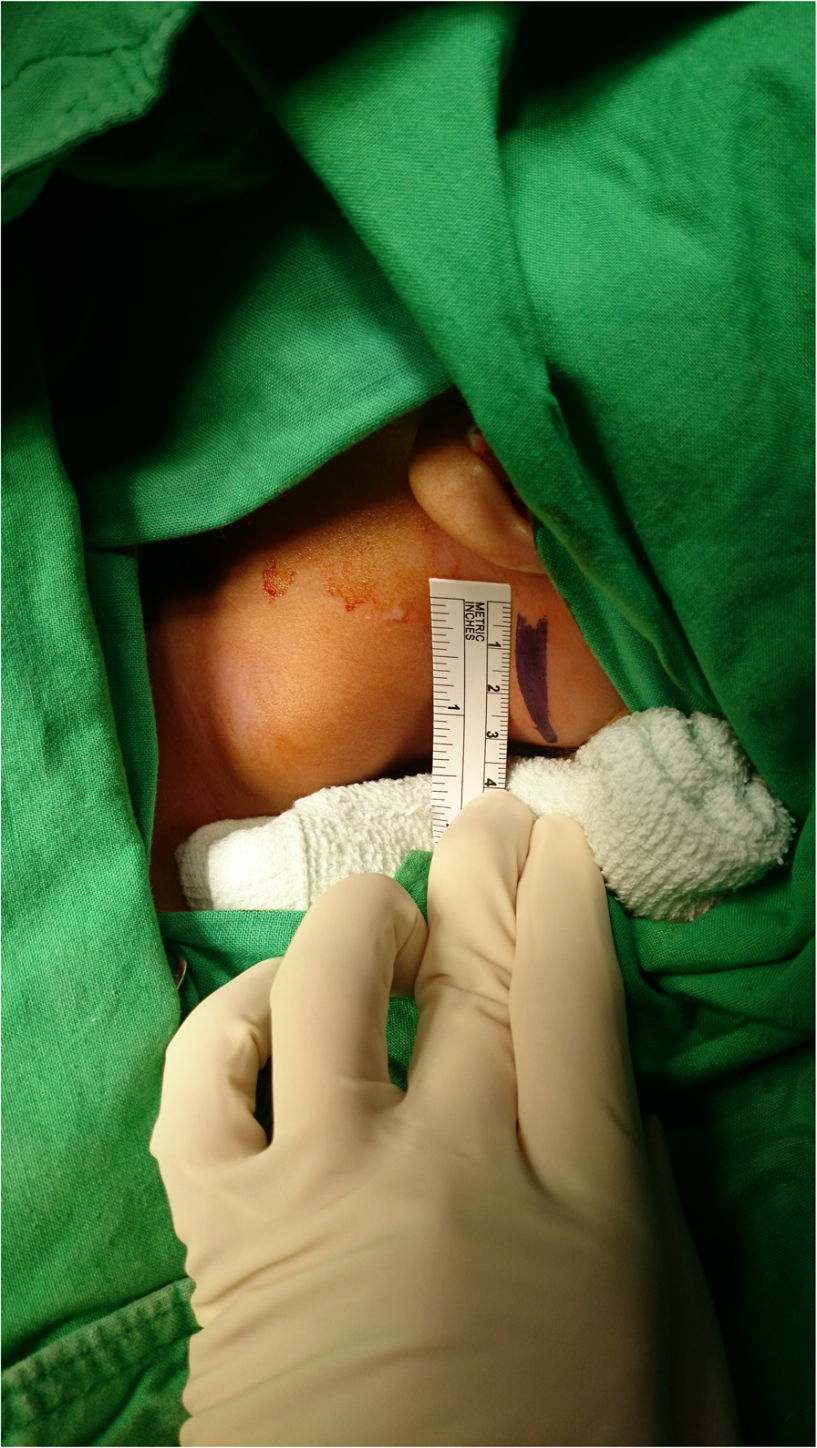
Intraoperative findings: retroauricular hairline approach with a 3-cm skin incision in the hairline and via 7-cm skin flap tunnel

**Fig. 3:**
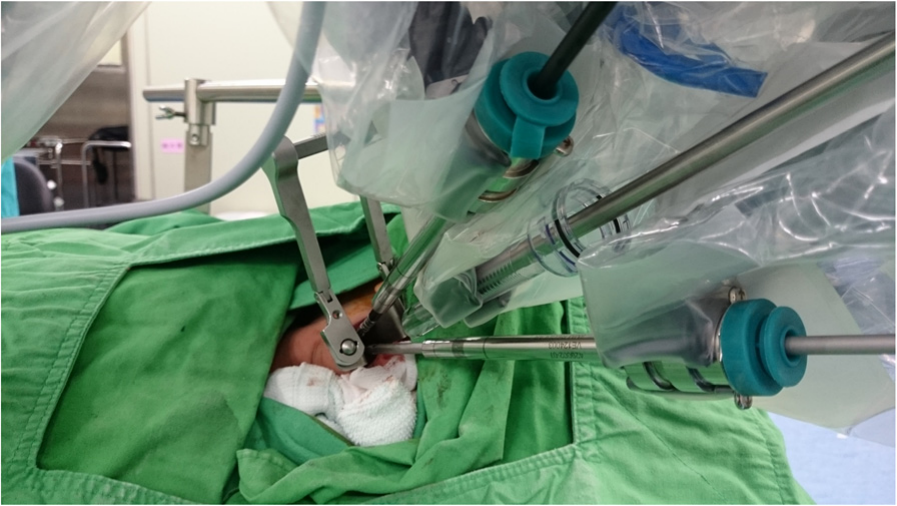
Robot-assisted excision via a small retroauricular incision was facilitated by the application of the Yang’s retractor

**Fig. 4:**
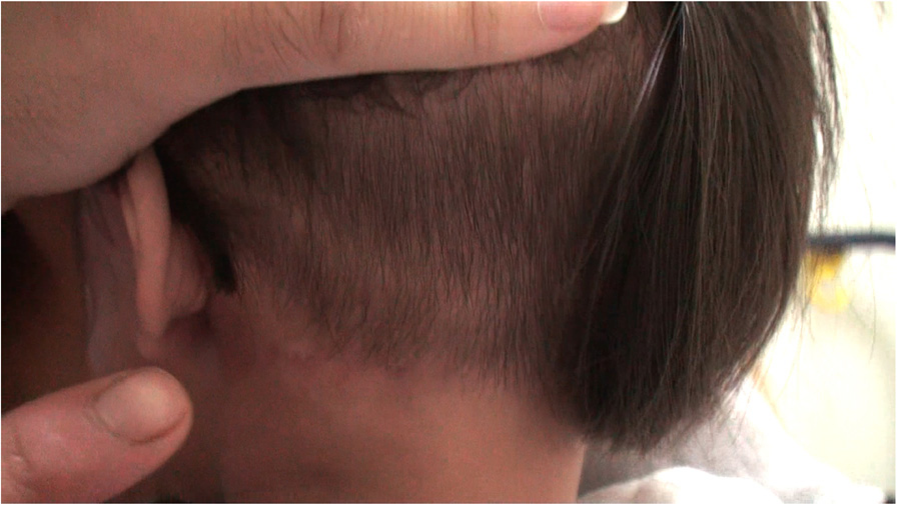
Follow-up visit 3 months postoperatively showed the incision scar well hidden in the hairline

A 19-month-old Asian baby girl visited the otolaryngology department for the evaluation of a sudden swelling in the left side of her neck, which had been found by her parents after a fall in the bathtub on the day of the outpatient clinic visit. Other than having a prominent swelling in the left side of her neck, the girl was asymptomatic. A physical examination showed a 4 × 4 cm sized cystic mass in the posterior triangle of the left side of her neck with a bluish hue over the neck skin. Over the course of 2 months of follow-up at the outpatient clinic, the left neck swelling decreased slightly to 3 × 3 cm in size with subsequent resolution of the bluish skin coloration. Subsequent computed tomography imaging of her neck showed a well-encapsulated cystic mass in the left side of her neck posterior to the sternocleidomastoid muscle (Fig. 1). To avoid leaving a visible scar in her neck, robot-assisted excision was performed via a small retroauricular hairline approach. A self-retaining retractor, the Yang’s retractor [3] was first applied through a 3-cm trans-hairline skin incision after the elevation of a 7-cm skin flap (Fig. 2). Robot-assisted excision was performed successfully via a 7-cm-long skin flap tunnel (Fig. 3, Additional file 1: Video 1). The mass showed the presence of a bloody-colored serous fluid. The final pathology report confirmed the diagnosis of cystic hygroma. The follow-up visit 3 months postoperatively showed no evidence of recurrence and the scar was well hidden in the hairline (Fig. 4).

## Discussion

Cystic hygromas represent 2.5–5 % of benign congenital cervical masses, generally appearing as a spontaneous growth of preexisting subclinical lesions [4]. In our case, the child developed a large neck mass after a fall in the bathtub. Trauma as an etiology for the development of cystic hygroma has been previously suggested, especially when it occurred in an adult [5]. We believe that the child probably had a preexisting lymphatic malformation and blunt trauma to the neck led to bleeding into the cystic cavity with the development of a noticeable cystic hygroma.

The treatment of cystic hygroma remains challenging for pediatric head and neck surgeons, especially when the parents are often concerned about the permanent cosmetic outcome. In addition, surgical excision carries the risk of damaging the surrounding nerves and blood vessels, scarring, and recurrence due to incomplete excision [6]. Other alternative treatment modalities developed with the intent of improving cosmetic outcome, or for patients who are poor candidates for surgery, including watchful waiting, sclerotherapy, radiation, hormone therapy, the transoral approach, and endoscope-assisted excision [4, 6, 7].

The robot-assisted excision using the retroauricular approach has recently been reported for branchial cleft cysts, which usually occur in an older age group between the ages of 10 and 50 years [8]. Compared to endoscopic surgery, robotic surgery has the advantages of a three-dimensional view, more flexibility with a wider range of motion, and tremor-free movements, which are very important in a limited working space in the neck area especially in the pediatric population.

## Conclusions

This present case reports a successful robot-assisted excision of cystic hygroma through a concealed and minimally invasive retroauricular hairline incision in a child less than 2 years of age. The innovative use of the Yang’s retractor helps to elevate the skin flap, maintain the working field, and provide space for introduction of surgical instruments of the robotic Da Vinci Si surgical system (Intuitive Surgical, Inc., Mountain View, CA, USA). With improvements in surgical techniques and instrumentation, the treatment of cystic hygroma is now feasible through a minimally invasive robot-assisted surgery, hence potentially improving not only the cosmetic outcome but also the life quality of these patients typically afflicted with the disease early in their lives. This is the first report in the medical literature describing robot-assisted excision via a small concealed hairline approach in the surgical treatment of cystic hygroma.

## Additional file

Additional file 1: Robot-assisted excision of cystic hygroma. (WMV 60 mb)
